# Metal-Based Nanoparticles: Antibacterial Mechanisms and Biomedical Application

**DOI:** 10.3390/microorganisms10091778

**Published:** 2022-09-03

**Authors:** Domenico Franco, Giovanna Calabrese, Salvatore Pietro Paolo Guglielmino, Sabrina Conoci

**Affiliations:** 1 Department of Chemical, Biological, Pharmaceutical and Environmental Sciences, University of Messina, Viale Ferdinando Stagno d’Alcontres, 31, 98168 Messina, Italy; 2 Department of Chemistry ‘‘Giacomo Ciamician’’, University of Bologna, Via Selmi 2, 40126 Bologna, Italy; 3 LabSense Beyond Nano, URT Department of Physic, National Research Council (CNR), Viale Ferdinando Stagno d’Alcontres, 31, 98168 Messina, Italy

**Keywords:** antibacterial mechanisms, bacterial resistance, biofilm, metal and metal oxide nanoparticles, nanotechnology, nanomedicine

## Abstract

The growing increase in antibiotic-resistant bacteria has led to the search for new antibacterial agents capable of overcoming the resistance problem. In recent years, nanoparticles (NPs) have been increasingly used to target bacteria as an alternative to antibiotics. The most promising nanomaterials for biomedical applications are metal and metal oxide NPs, due to their intrinsic antibacterial activity. Although NPs show interesting antibacterial properties, the mechanisms underlying their action are still poorly understood, limiting their use in clinical applications. In this review, an overview of the mechanisms underlying the antibacterial activity of metal and metal oxide NPs will be provided, relating their efficacy to: (i) bacterial strain; (ii) higher microbial organizations (biofilm); (iii) and physico-chemical properties of NPs. In addition, bacterial resistance strategies will be also discussed to better evaluate the feasibility of the different treatments adopted in the clinical safety fields. Finally, a wide analysis on recent biomedical applications of metal and metal oxide NPs with antibacterial activity will be provided.

## 1. Introduction

The excessive and improper use of antibiotics, causing antimicrobial resistance, has become one of the most significant problems in the clinical field in recent years [1].

From the first documented case [2] to date, we are observing a growing increase in bacteria resistant to common antibiotics, with the consequent spread of so-called multi-drug-resistant (MDR) strains [3]. The Centers for Disease Control and Prevention (CDC) have estimated that over 2 million infections and 23,000 deaths associated with MDR bacteria occur annually [1]. These pathogenic strains belong to the group named ESKAPE and include *Enterococcus faecium, Staphylococcus aureus, Klebsiella pneumoniae, Acinetobacter baumannii, Pseudomonas aeruginosa* and *Enterobacter* species [4].

Antibiotic resistance is probably due to the misuse and overuse of antibiotics, as well as poor infection prevention and control [5]. In the literature, several antimicrobial resistance mechanisms are reported, including: (i) limiting the uptake of a drug; (ii) modifying a drug target; (iii) inactivating a drug; and (iv) active drug efflux [6]. These mechanisms, that may be native or acquired, allow the survival of the resistant strains and their spread, with the consequent failure of antibacterial therapies.

Gram-negative and Gram-positive bacterial strains exhibit different mechanisms of antimicrobial resistance, mainly due to differences in cell wall structure. Specifically, while Gram-negative bacteria limit the uptake of a drug, Gram-positive bacteria, missing LPS in the outer membrane, inactivate the drug [7,8].

In recent years, several strategies to overcome antimicrobial resistance have been developed in order to obtain new broad-spectrum drugs with a better half-life [9]. Among these approaches, the possibility of using nanomaterials as new non-traditional antimicrobials agents has been investigated. In vitro studies have demonstrated that nanomaterials exhibit toxic effects against several bacterial strains, suggesting their feasibility for biomedical applications, including drug delivery and tissue engineering [10,11,12]. Among the most promising nanomaterials are metal and metal oxide nanoparticles (NPs). 

The antibacterial activity associated with metal NPs has long been known; however, their mechanisms are still poorly understood. In recent years, several antibacterial mechanisms, ascribable to both physical damage and chemical interactions, have been proposed, including ion leaching, ion dissolution, and/or reactive oxygen species (ROS) production, involving the loss of cell wall and cell membrane integrity and, consequently, the interference with some metabolic pathways essential for bacterial viability [13,14]. 

Furthermore, some requirements are crucial for the antibacterial activity of metal NPs, such as size, shape, surface/volume ratio and surface functionalization, mainly affecting the biocompatibility and bactericidal properties. In this context, nanotechnology represents a powerful tool to design NPs with appropriate physico-chemical properties in order to reduce their cytotoxic effect and the risk related to their use in biomedical applications [15,16,17,18].

This review aims to provide an overview of metal NP antibacterial mechanisms related to different bacterial species, higher microbial organizations (biofilm), and the physico-chemical properties of NPs themselves. In addition, bacterial resistance strategies are also discussed to better evaluate the feasibility of different treatments adopted in the clinical and human safety fields. Finally, a wide analysis of recent biomedical applications of antibacterial NPs will be provided.

## 2. Gram-Positive and Gram-Negative Bacteria and Biofilm

Although metallic NPs have demonstrated broad-spectrum antibacterial properties, the composition and organization of the bacterial cell wall can influence their bactericidal efficiency. In addition, higher microbial organizations such as biofilm can hinder the diffusion, subsequently increasing the lethal dose compared to planktonic cells.

### 2.1. Gram-Positive and Gram-Negative Bacteria 

**Figure 1:**
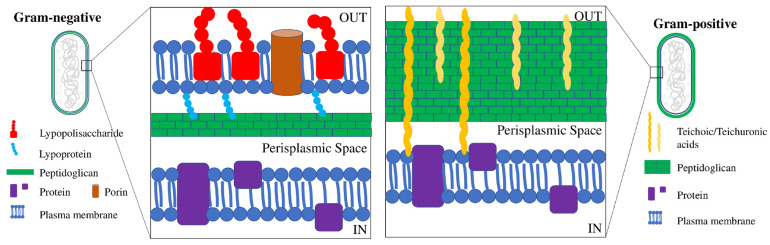
Schematic structure of Gram-negative and Gram-positive cell walls. Gram-negative bacteria have an inner and an outer cell membrane (double lipid bilayer) and only a thin layer of peptidoglycan in the periplasmic space, whereas Gram-positive bacteria show only one lipid plasma membrane and a thick peptidoglycan layer interlinked with teichoic and lipoteichoic acids.

Except for a few species, most bacteria have an outer protective cell wall and membrane and, depending on the cell wall composition, they can be classified as “Gram-positive” or “Gram-negative”. Gram-positive and Gram-negative bacteria can be differentiated by Gram-staining (Figure 1).

Gram-positive bacteria contain a uniform and relatively thick (20–80 nm) wall, consisting of numerous layers of peptidoglycan, crisscrossed by teichoic and teichuronic acids (polymers of alcohols and phosphates). As a result, they have a very polar structure that only allows the permeation of hydrophilic compounds. On the other hand, Gram-negative bacteria contain a very thin (<10 nm) and complex wall, in which peptidoglycan is surrounded by an outer membrane containing an internal phospholipid sheet and an external lipopolysaccharide (LPS). The presence of the outer membrane makes the wall highly selective, also hindering the passage of small hydrophilic molecules, including water [19,20]. Since Gram-negative and Gram-positive bacteria display a different structural organization, they respond differently to antimicrobial agents, including metal NPs. Several studies, in fact, have demonstrated that Gram-positive bacteria are more resistant to NPs than Gram-negative bacteria, because their different arrangement and structure facilitate the entrance of released ions [21,22,23,24,25,26]. Furthermore, it has also been demonstrated that metal NPs can interact with the repeated units of amino acids and carbohydrates of the peptidoglycan layer. Therefore, since Gram-positive walls contain a greater amount of peptidoglycan than Gram-negative walls, Gram-positive bacteria have a higher resistance to damage [27,28]. For the same reasons, Gram-positive bacteria are considerably more resistant to damage by metal ions, due to their trapping by negatively charged peptidoglycan [24]. In addition, it has been reported that the different susceptibility of Gram-negative bacteria to NPs could also be due to the presence of LPS molecules, which increase the negative charge of the cell membrane compared to Gram-positive bacteria [29]. 

Recent evidence has also suggested that the mosaic structure of LPS molecules in Gram-negative bacteria make some regions more negatively charged, and thus NPs (positively charged) tend to aggregate in these areas, leading to a localized toxic effect [30]. Differently, ROS produced outside the bacterial cell induce the same antibacterial activity in both Gram-positive and Gram-negative bacteria. This can be attributed to the fact that these radicals, mainly hydroxyl radicals, are negatively charged and so can hardly penetrate into the cell membrane [31]. 

### 2.2. Biofilm

**Figure 2:**
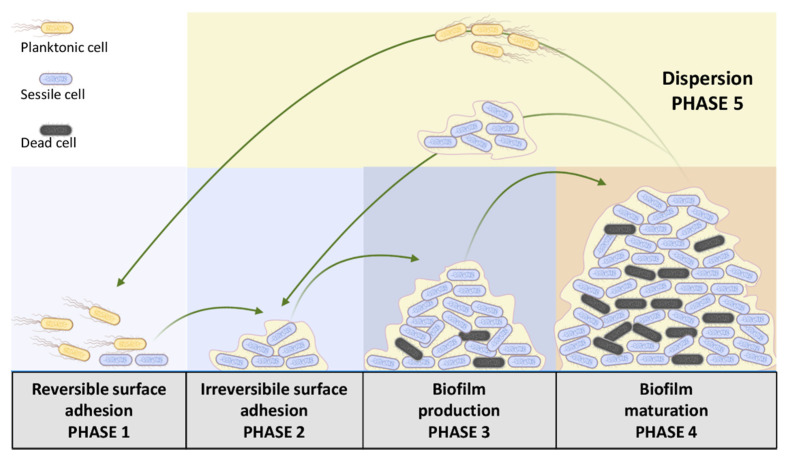
Schematic representation of cyclic biofilm formation, from reversible adhesion of bacteria to biofilm dispersion, consisting of the evacuation of matrix–bacteria aggregates.

Microorganisms are able to organize themselves into a higher life structure and grow adhered to almost every surface, forming architecturally complex communities, termed biofilms [32]. These structures consist of bacteria incorporated in an extracellular polymer matrix (polysaccharides, proteins, glycopeptides, nucleic acids, and lipids) whose primary function is to protect bacteria from all unfavorable chemical–physical agents, including antibiotics [33]. Figure 2 reports the several steps of biofilm synthesis and maturation [34]. 

The process starts with the transition from reversible surface adhesion (phase 1) to the irreversible one (phase 2). In this transition, some genetic pathways are blocked and replaced with those related to the production of the main components of the biofilm matrix (phase 3) and its subsequent maturation (phase 4). The final step of the cyclic biofilm formation includes the dispersion of both planktonic cells and full bacteria–matrix aggregates (phase 5). Recent evidence has shown that biofilm-like aggregates can also form in the absence of surfaces and attach at a later time [35]. Since the extracellular matrix acts as a filter, the antibacterial success of NPs depends almost exclusively on their interactions with this polymeric matrix. In this context, NP–matrix interactions include three consequential phases that lead to the destruction of the extracellular matrix and bacteria killing: (i) NPs’ adhesion to the matrix, (ii) NPs’ inclusion within it, and (iii) lethal interaction between bacteria and NPs. Although antibacterial mechanisms of metal NPs are identical to those on planktonic ones, biofilms offer greater resistance by limiting the diffusion of NPs and their contact with biofilm cells [36,37,38]. Surface-covering strategies using NPs can act on the initial stages of bacterial adhesion, effectively providing an excellent biofilm inhibition strategy. An example is represented in the surface coating of AgNPs, which is able to prevent biofilm formation from both Gram-negative and Gram-positive bacteria [39,40]. Similar results were also obtained with the zinc oxide (ZnO) NP coating [41,42].

## 3. Influence of Metal NPs’ Physico-Chemical Properties on Antibacterial Activity 

Physico-chemical features of metal NPs are critical factors for their antimicrobial and antibiofilm activity. Heavy metals, with densities greater than 5 g/cm^3^, have excellent antimicrobial activity [43]. At the nanoscale, the physical and chemical properties of metals change dramatically from that of the bulk material due to size and shape effects, as well as the high surface area to volume ratio of nanomaterials [44]. Consequently, several properties such as ion release, hardness, plasmon and super-paramagnetism are affected [45,46,47]. Moreover, the response to external stimuli—such as light in the case of photocatalytic and photothermal activity [48,49], and magnetism in the case of magnetically induced hyperthermia activity [50,51]—is modified compared to the bulk metal. 

### 3.1. Size 

Typically, smaller NPs have higher antibacterial activity due to their ability to penetrate a cell and to inhibit bacteria growth [52,53,54,55,56,57,58]. Moreover, smaller NPs have a relatively large surface area/volume ratio compared to larger NPs, favoring ROS production. Skomorokhova et al. tested the antibacterial activity of AgNPs of three different sizes against *Escherichia coli* in their study, demonstrating that larger particles were less effective as antibacterial agents than smaller NPs, and that the antibacterial activity for particles of the same size was strictly dose- and time-dependent [59]. Similarly, Korshed et al. found that the bactericidal effects were inversely correlated to the NPs’ size [60]. Furthermore, they showed that smaller AgNPs induced more ROS production than larger AgNPs. In another study, it was shown that AgNPs of 18 nm in size were more toxic than those of 80 nm in water, but their toxicity declined to a similar level in the PBS buffer [52]. Zare et al. synthesized ZnONPs of various sizes and morphologies and found that these NPs displayed very good antibacterial and antioxidant activity that was size- and morphology-dependent [61]. Adams et al. found that palladium NPs (PdNPs) with smaller diameter, i.e., 2.0 ± 0.1 nm, exhibited higher toxicity relative to the 2.5 ± 0.2 nm and 3.1 ± 0.2 nm NPs. Furthermore, they demonstrated that Gram-negative *E. coli* required higher concentrations and longer exposure times of PdNPs to inhibit bacteria growth compared to the Gram-positive *S. aureus* [62]. 

### 3.2. Shape

NPs can exhibit different shapes, including spherical, dots, wires, rods, stars, flowers and 2D materials, affecting their antibacterial activity [16,63,64,65,66]. Although NPs’ spherical shape is the most common, nanocubes and nanorods are more effective than other shapes, possibly due to the exposed planes and to the oxidation levels of the metals. Specifically, it has been suggested that less stable planes require less energy to form oxygen vacancies, linking the bactericidal activity of the NPs to the stability of the planes [14]. Wang et al. evaluated CeO_2_ and Ag/CeO_2_ NPs with different shapes and demonstrated that nanocubes and nanorods displayed higher bactericidal activities than other shapes due to their exposed crystal planes and oxidation ability [14]. Hong et al. showed that Ag nanowires displayed a lower antibacterial activity compared to Ag nanocubes and nanospheres, due to their lower interaction with bacterial cells [63]. The same authors found that silver nanocubes had better antimicrobial properties than nanospheres, due to the larger contact areas and higher reactive facets. In their paper, Franco et al. developed a magnesium–hydroxyapatite (Mg-HA) scaffold functionalized with Au nanorods (AuNr) and showed that the presence of AuNr induced 100% bacteria reduction after 24 h for both *S. aureus* and *E. coli* [16].

Furthermore, it has been also observed that the presence of corners, edges or defects increases NPs’ toxicity, probably due to the increased surface area, affecting their adsorption, binding, or ROS production. Huynh et al. found that gold nanostars can be used instead of antibiotics in acne treatment due to their strong bactericidal activity against *Propionibacterium acne* [64]. Silver nanoflowers coupled with a low dose of antibiotics led to the complete eradication of drug-resistant bacteria, probably due to the release of ROS and antibiotic uptake induction mainly mediated by NPs [65]. This evidence suggests that the shape of NPs has a crucial role in affecting antimicrobial properties.

### 3.3. Surface Charge 

Another important factor for NPs’ antibacterial activity is given from their surface charge. It has been shown that NPs with positive charge display higher toxicity due to their electrostatic interaction with the negative charge of the bacterial cell wall [29,55,67,68,69]. Li et al. showed that magnetic NPs with a positive charge (NP+) efficiently attracted over 90% of *E. coli*, while negatively charged magnetic NPs (NP−) did not show any affinities. These results suggest that NPs+ have good potential to capture bacteria via electrostatic attraction [68]. Abbaszadegan et al. compared the antibacterial efficiency of three different AgNPs that were positively, negatively, and neutrally charged. They found that positively charged NPs showed the highest bactericidal activity, while the negative ones showed the lowest [29]. El Badawy et al. evaluated the toxicity of four AgNPs with several surface charges, ranging from highly negative to highly positive. Their results showed that AgNPs exhibited surface charge-dependent toxicity on the bacterial species investigated [52,70]. In another study, it was demonstrated that positively charged Ag–polyethylenimine (BPEI) NPs tightly adhered to the bacterial surface, while no attachment was observed for negatively charged citrate–Ag NPs [55]. In contrast, Agnihotri et al. found that negatively charged AgNPs (citrate-stabilized) exhibited a strong antibacterial activity against *E. coli* and *S. aureus*. Their results also indicated that the antibacterial efficacy increased with lowering particle size [71].

## 4. Antibacterial Mechanisms 

In this section, the antibacterial mechanisms of metal NPs including (i) physical interactions, (ii) ion leaching and dissolution, and (iii) ROS production will be analyzed. 

### 4.1. Physical Interactions

**Figure 3:**
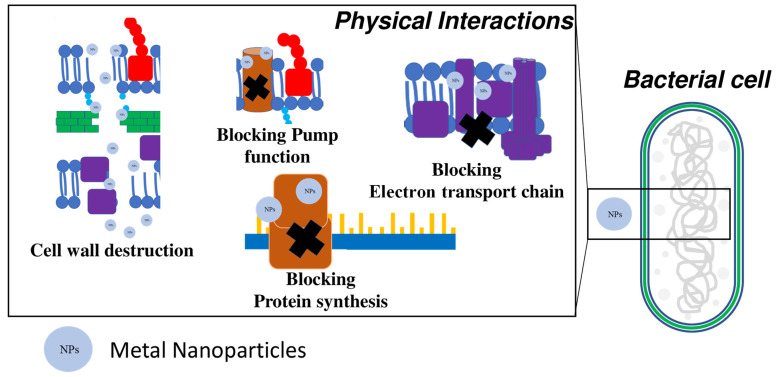
Schematic description and targets of antibacterial mechanisms due to a physical interaction mediated by metal nanoparticles (NPs).

It has been already demonstrated that metal nanomaterials can interact physically with the cell membrane/wall or with intracellular components [72] (Figure 3).

The cell wall has an important role in isolating cytoplasmic inclusions from the external environment and in hosting important metabolic pathways (e.g., the electron transport chain and controlled passage of molecules to and from the outside), so its destruction is fatal for bacterial cells [73]. Since the cell wall of both Gram-positive and Gram-negative bacteria is negatively charged, electrostatic interactions with positively charged NPs are favored.

The contact of bacteria with NPs lead to membrane damage due to their adsorption and penetration into the cell [56]. NPs’ adsorption causes cell wall depolarization, modifying the negative charge of the wall and making it more penetrable. Consequently, the cell wall is destroyed, and ROS are produced [74].

Several studies have shown that AgNPs adhere to the cell wall, degrading it and increasing ion passage towards the cytosol [54,75]. Ninganagouda et al. demonstrated that AgNPs are able to anchor on bacterial surfaces, causing death via ruptured cell membranes and the leakage of intracellular components [76]. Other researchers have demonstrated that MgONPs and Mg(OH)_2_NPs can cause cell death by electrostatic adsorption onto the cell wall without entering the cell [77,78]. 

The cellular uptake of NPs is another mechanism associated with physical interaction, and occurs when NPs are small enough to cross the cell membrane [79]. Mukha et al. showed that the antibacterial activity of AgNPs smaller than 10 nm is due to membrane damage and their penetration into the cell [80]. Similarly, Dong et al. evaluated NPs with different sizes and concluded that AgNPs with smaller size are more effective because they are able to cross the cell membrane [81]. Oves et al. synthesized AgNPs with bacterial exopolysaccharides, spherical shape, and a size of about 35 nm. They demonstrated that the antimicrobial activity of these NPs against *B. subtilis* and *methicillin-resistant Staphylococcus aureus* (MRSA) is due to ROS production inside bacterial cells. Furthermore, they showed that NPs also had excellent properties against biofilm formation [82]. 

In another study, it was demonstrated that the bactericidal activity of gold (Au) NPs against *E. coli* was due to the inhibition of ribosome subunits, in addition to the alteration of membrane and ATPase activities [83].

### 4.2. Ion Leaching and Dissolution

**Figure 4:**
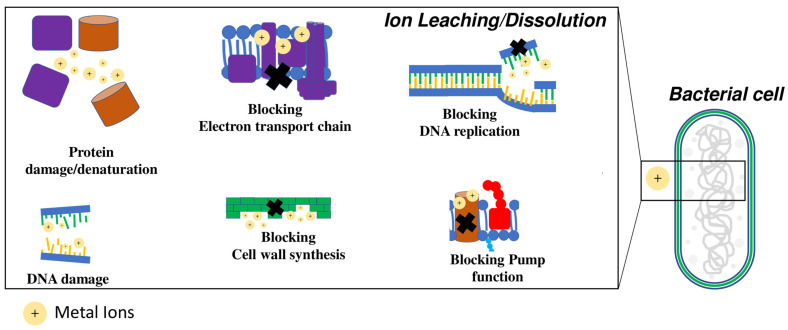
Schematic description and targets of antibacterial mechanisms due to the release of metal ions (+) by nanoparticles (NPs).

The antibacterial activity of NPs can be also attributed to ion leaching; these ions can diffuse inside the cell and interact with the cellular membrane and wall, as well as cell macromolecules such as proteins and nucleic acids [84,85,86,87] (Figure 4). Several studies have demonstrated that environmental conditions, including pH and NP dissolution rate, can significantly affect the antimicrobial activity of NPs themselves. Saliani et al. showed that the inhibition of bacterial growth due to ZnONPs increased when pH decreased from 7 to acidic pH [88]. Analogously, Moreau et al. observed an increased dissolution of ZnONPs under acidic conditions, suggesting a higher release of Zn^2+^ ions [89]. Pereziazhko et al. suggested that once released into the aquatic system, AgNPs undergo oxidative dissolution which results in the release of Ag + ions and the induction of antibacterial activity [90]. In addition, they observed that the size-dependent dissolution of AgNPs was larger in acetic acid than in water. 

Wigginton et al. showed that over 50% of bacterial proteins have a strong binding affinity for NPs and metal ions, including enzymatic and non-enzymatic proteins [91]. Therefore, bacterial cells undergo damage both at the metabolic level and at the structural level, due to the loss of cellular integrity and the energy transport chain. 

The NPs’ interaction with nucleic acids is certainly the main antibacterial mechanism due to the ion diffusion in the cells. Chatterjee et al. reported that *E. coli* and *S. aureus* after AgNP treatment showed DNA condensation. Furthermore, their results indicated that the DNA of *E. coli* was more susceptible compared to *S. aureus* [92]. Another study indicated that the accumulation of high concentrations of metal ions can lead to double-stranded DNA breakdown [93]. 

Ions released from metal NPs can be significantly influenced by the medium in which they are dispersed [94]. Levard et al. found that chloride present in seawater or culture medium can favor ion release from AgNPs [95]. This release, despite leading to an initial increase in bactericidal activity, reduces the NPs’ life and, consequently, their antimicrobial activity overtime. The same effect of bactericidal activity reduction is also due to oxidation and the interaction of zero-valent iron NPs with natural organic matter in aerobic conditions [96]. 

### 4.3. Production of Reactive Oxygen Species

**Figure 5:**
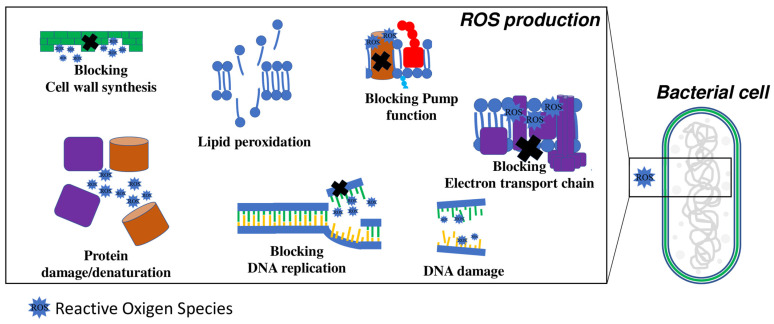
Schematic description and targets of antibacterial mechanisms due to the ROS produced by nanoparticles (NPs).

The production of ROS is another important mechanism involved in metal NP-mediated antibacterial activity (Figure 5).

ROS can disrupt bacterial cells if they are produced either inside or outside the cell [14,97]. High ROS concentrations can damage the cellular membrane [98,99] by oxidative stress and lipid peroxidation, and the wall by the breakdown of the peptidoglycan structure [55,100,101], degrading proteins and nucleic acids [102] and thus leading to cell death. Metal nanomaterials increase ROS production in bacterial cells that cause DNA denaturation by intercalation between the purine and pyrimidine bases [99]. Consequently, metabolic and transduction signals are altered, and the cell growth is inhibited. The most common metal nanomaterials that produce antibacterial activity mediated by ROS production are silver, zinc oxide, and titanium dioxide [98,103,104,105]. The ROS amount induced by metal nanomaterials depends on the size and chemistry, while the effect of shape is unknown. Mujeeb et al. found that silver–copper nanocomposites (Ag-CuNCs), synthetized using an *Olax scandens* leaf extract showed a greater antimicrobial activity than monometallic AgNPs with an increase in ROS production [106]. Wang et al. found that the bactericidal activity of Ag/CeO_2_ NPs in *E. coli* is mainly due to intracellular ROS production as well as cell wall and membrane disruption [14], and not due to the ion silver release.

### 4.4. Bacterial Resistance Strategy against NPs

Bacteria can regulate their electrical surface charge to repel NPs with different charges, modifying the structure of the phospholipidic membrane or cell wall [107]. Gram-positive bacteria can reduce their negative surface charge by the incorporation of D-alanine into the cell wall [108]. Otherwise, Gram-negative bacteria modify their LPS in the outer membrane by adding phosphoethanolamine to lipid A [109]. 

The activity of bacterial cells on NPs can modify their physico-chemical properties, including aggregation and ligation, redox reactions, biomacromolecule adsorption altering ion release and, consequently, antibacterial activity. 

It is known that resistance to ion release can be favored by bacterial efflux systems. These systems involve several protein families belonging to resistance-nodulation-cell division proteins (membrane fusion protein family or outer membrane factors), cation diffusion facilitators, and members of P-type ATPases [110,111]. Otherwise, metal ions can be intra- and extracellular-sequestered, or reduced by species-specific pigments [112,113]. Ellis et al. observed that phenazine pigments, including pyocyanin, pyochelin, and pyoverdine, can induce resistance by reducing Ag^+^ in Ag^0^ in *P. aeruginosa* and its biofilm [113]. These systems are controlled by a metal regulatory and homeostasis system and can be codified by specific operons present in bacterial DNA, or plasmids [114,115]. It has recently been observed that exposure to sub-lethal doses of metal NPs increases the conjugative transfer rate, favoring the co-selection and co-expression of antibiotic resistance genes [116,117].

Recently, it has been shown that Gram-negative bacteria, such as *E. coli* and *P. aeruginosa*, produce extracellular molecules, such as flagellin, able to favor AgNPs’ aggregation and, consequently, to reduce antibacterial activity on both planktonic and biofilm cells [118,119]. NPs’ size is also important for their diffusion in biofilm through existing pores and water channels [15,120]. Choi et al. demonstrated that biofilms are more resistant to nanosilver inhibition than planktonic cells, suggesting that this resistance could be at least partially due to NPs aggregation and retarded Ag ion/particle diffusion [36]. 

## 5. Antimicrobial Application of Metal NPs

### 5.1. Biomedical Application

Due to increasing drug-resistant bacteria, metal NPs have attracted growing interest in several biomedical applications as antibacterial agents. NPs exhibit several advantages such as easy production and the possibility to modulate their antibacterial effects by changing physical parameters. 

One of the most common uses of NPs is in implantable devices. Implantable devices must possess specific requirements, including good biocompatibility, tissue affinity, corrosion resistance and, in particular, antibacterial property [121]. 

Recently, several approaches have been proposed to improve the antibacterial effects of implants by coating with several metal and metal oxide NPs [122,123,124,125,126,127]. Poly(methyl methacrylate) (PMMA)-based bone cement with AgNPs showed a significant reduction in surface colonization and biofilm formation [128]. Similarly, Kose et al. used titanium nails coated with silver-doped hydroxyapatite for arthroplasty surgery, observing good antibacterial activity, the absence of inflammation around the prostheses, and no cytotoxic effect due to the silver ions [129]. 

Significant antibacterial results have also been found in other medical devices, such as catheters and dental implants. Some researchers found that functionalization with NPs reduced bacterial growth and biofilm production after catheter implantation [130,131]. Chen et al. optimized a AgNP-coated collagen membrane for dental implants, showing excellent antibacterial effects both against *S. aureus* and against *P. aeruginosa,* with low cytotoxicity [132]. Ramazanzadeh et al. coated dental plaque chalk with copper and zinc oxide, showing significant antibacterial activity against *Streptococcus mutans* from 6 to 24 h [133]. 

NPs are used also as antibacterial additives in dressings for the treatment of skin wounds. Both Gram-positive and Gram-negative pathogenic bacteria can lead to chronic infections associated with skin wounds. AgNPs significantly inhibited bacterial growth and increased the rate of wound healing when used in combination with poly(vinyl alcohol) and chitosan [134].

Finally, NPs also find application in the agro-food field, especially in food packaging, where their use in the detection and killing of pathogens is closely linked to human health [135,136].

### 5.2. Recent Application of Metal NPs

The following sections will discuss the latest evidence on the antimicrobial activity of some metal NPs in several applications.

*Silver-based nanoparticles:* AgNPs and Ag_2_ONPs are used in a wide range of applications, such as dentistry, scaffolds for tissue engineering, wound healing materials, food packaging, drinking water disinfection, and textile manufacturing, due to their high antibacterial, antifungal and antiviral activities [137,138,139,140,141,142,143,144,145].

It has been observed that AgNPs incorporated into crystallized hydroxyapatite (HA) or titanium scaffolds have significant antibacterial activity against both Gram-positive and Gram-negative bacteria [18,146,147], and show improvement to bone healing [148]. Shimazaki et al. implanted, in rat models, Ag-HA-coated titanium plates to test their activity against methicillin-resistant *Staphylococcus aureus* (MRSA) compared with HA-coated plates. Their results showed a lower number of MRSA colony-forming units (CFUs) on the Ag-HA-coated plates, suggesting the promising use of the coating in clinical applications [149]. Similarly, Saravanan et al. reported that chitosan/nanohydroxyapatite-containing AgNPs exhibited antimicrobial activities against Gram-positive and Gram-negative bacteria, with negligible cytotoxicity versus rat osteoprogenitor cells and human osteosarcoma cells [150]. 

Alt et al. performed an in vitro study comparing nanosilver-loaded bone cement with gentamicin-loaded and plain cement against *S. epidermidis*, methicillin-resistant *Staphylococcus epidermidis* (MRSE), and MRSA. Their results showed that only the nanosilver cement had good antibacterial efficacy against both bacterial strains [151].

In vitro and in vivo studies have evaluated the utility of nano-Ag as an additive to prevent infection with *Porphyromonas gingivalis* [152,153,154] or to improve antibacterial surface coating against *S. aureus* and *E. coli* [155]. AgNPs have also been used in the preparation of catheters to improve their inhibitory effects against infection [156,157,158], especially due to MRSA, and are able to form biofilms and bacterial dispersion [159]. Wilkinson et al. reported that dressings containing AgNPs exhibited a higher antimicrobial effect than bulk silver due to the sustained release of silver ions on the exudate released from the wound [160]. AgNPs’ antibacterial activity is due to their physical interaction with the peptidoglycan cell wall of bacteria, causing structural changes that increase membrane permeability and subsequently cell death [161]. AgNPs also interact with exposed sulfhydryl groups of bacterial proteins to prevent DNA replication [162]. Silver oxide NPs (Ag_2_O) interact with the DNA of bacteria causing the loss of their replication ability. The cell cycle halts at the G2/M phase due to DNA damage, and the cells are subsequently killed by oxidative stress [72]. Moreover, AgNPs with a size smaller than 6 nm can penetrate the entire depth of biofilm [163].

*Gold-based nanoparticles*: The incorporation of AuNPs in PMMA-based bone cement improves the mechanical properties of the polymeric matrix and reduces the formation of *S. aureus* biofilms [164]. The decrease in size of AuNPs to the nanocluster (NC) range (1–2 nm in diameter) has been found to enhance antimicrobial activity against Gram-positive and Gram-negative bacteria, mainly due to increasing ROS levels, maintaining a low cytotoxic and genotoxic effect on the host cells [165]. 

In addition, it has been suggested that alteration of the shape (from NPs to nanospikes or nanorods) leads the expansion of the absorption wavelength to the near-infrared region, enhancing light-to-heat conversion [166]. Such nanostructures have potential applications in biomimetic scaffolds [16,167], water sterilization, and photothermal/photodynamic therapy [168,169]. In vivo studies have shown that both AuNPs and AuNCs exhibit toxicity by decreasing red blood cell count and damaging organs such as the spleen, liver and kidney [170]. 

The main advantages related to gold-based nanostructures are rapid and simple preparation and their use as a bio-conjugable platform. In addition, the ability to absorb, scatter, and enhance the electromagnetic field of light makes them particularly versatile in both diagnostic and therapeutic systems.

*Copper-based nanoparticles*: The antimicrobial activity of copper NPs (CuNPs) is comparable to that of AgNPs, although bactericidal efficacy against Gram-negative strains is lower [171,172]. Similar to other metal NPs, smaller sizes exhibit a greater bactericidal effect, due to an increase in surface/volume ratio and a closer interaction with membrane and bacterial wall structures [173]. CuNPs are used in several medical applications, such as burn treatment, antibacterial prostheses, catheters, vascular grafts and teeth [174]. CuNPs have been embedded within dendritic polyglycerol to form coatings with a high antibacterial efficacy against both Gram-negative and Gram-positive strains, as well as antibiotic-resistant ones [175].

The antibacterial mechanism has been described as “attract–kill–release” by both ROS production and Cu^2+^ release, with long-term and good biocompatibility properties. Other applications of CuNPs involve their encapsulation in alginate beads, or their use as a coating for polyurethane foam for the development and design of antibacterial water filters [176]. The advantage of using CuNPs compared to other metal NPs, such as AgNPs and AuNPs, is the low production cost. Furthermore, NPs with high antibacterial and anticancer power have been obtained by green technology, reducing cytotoxic side effects [177].

*Iron-based nanoparticles*: Zero-valent iron NPs without polymeric coatings have been widely used for environmental remediation due to their ability to detoxify many contaminants by redox reactions. However, in vitro studies found toxicity toward terrestrial and aquatic biota, probably due to the oxidative stress and Fe^2+^-mediated cell membrane disruption [178,179]. The antibacterial mechanism of iron oxide is mainly due to the production of ROS, chlorosis, and hypoxia [179,180]. Iron oxides consist of magnetite (Fe_3_O_4_) and its oxidized forms, maghemite (γ-Fe_2_O_3_), and hematite (α-Fe_2_O_3_) [181], which are easily obtainable by both chemical–physical and green methods. Fe_3_O_4_ NPs penetrate the Gram-negative bacteria through the siderophore channels on the outer membrane. These NPs may be utilized as “Trojan horses” for transferring antibiotics coupled to them, and are usually blocked by the outer membrane into the Gram-negative bacteria [182]. Significant results were also obtained against biofilm. Ali et al. showed that α-Fe_2_O_3_ NPs interact with biofilm extracellular polymeric substances (EPS) and penetrate into cell, inhibiting growth by intracellular ROS formation [183]. Similarly, Ikuma et al. displayed that the deposition of α-Fe_2_O_3_ NPs on the polysaccharides of EPS is governed by electrostatic forces, so antibiofilm activity is mainly due to the direct contact between NPs and bacteria surface (contact-killing) [181]. Iron oxide NPs have been used as dental irrigants for the antibacterial treatment of the root canal, showing a significant reduction in *Entercoccus faecalis*, which is particularly resistant to treatment mediated by the activation of H_2_O_2_ [184]. α-Fe2O3 with bactericidal ability against Gram-negative and Gram-positive bacteria has been synthetized by pulsed laser ablation and coprecipitation methods [185]; γ-Fe_2_O_3_ has been also proved to be effective in bactericide activity [119], while similar results have been obtained from Fe_2_O_3_ obtained by an eco-friendly method on biopolymer templates via thermal decomposition [186]. Like other NPs, for iron oxide, coupling to other metals, such as silver [187], cobalt [188], copper [189,190], and bismuth [191], allows the obtainment of properties that can be used in different applications. Crystal hybrid structures based on ferrite NPs with Co or Cu showed a high bactericidal and fungicide activity [190,192]. Similar results against *S. aureus* have been found with bismuth, in hybrid nanorod-like structures [191]. On the other hand, peculiar characteristics of iron, such as magnetic properties, increase the possibility of its use in bacterial detection [193].

*Titanium*-*based nanoparticles*: Titanium NPs (TiNPs) are extensively used as additives in oral drugs, and are mixed with commercial and non-commercial dental resins and food-related products [194,195]. TiO_2_ NPs have also been used for the nanofunctionalization of titanium biomimetic scaffolds for bone tissue engineering. Calabrese et al., in their in vitro study, showed that titanium scaffold nanofunctionalized with TiO_2_ (Ti_TiO_2_) exhibited a good antibacterial activity towards *S. aureus*, reducing the number of CFUs by 99.4%. Furthermore, they showed that Ti_TiO_2_ increased the cell proliferation of human adipose-derived mesenchymal stem cells by 4.3-fold compared to the control titanium scaffold [125]. As a wide-bandgap semiconductor, TiO_2_ NPs have been incorporated into antibacterial paints or coatings for hospital touch surfaces. The excitation of TiO_2_ NPs by ultraviolet light produces ROS that can destroy many organic molecules [196]. This photocatalytic process can kill many classes of microorganisms. For antibacterial activity, photocatalysis triggers the downregulation of bacteria genes/proteins associated with regulatory, signaling, and growth functions [197]. Additionally, hybrid silver and TiO_2_ nanotubes activated by ultraviolet light or visible light range have been demonstrated to develop ROS in the form of hydroxyl radicals (OH•) and superoxide anions (•O_2_^−^) [198]. Under ultraviolet light, the photocatalytic activity of the TiO_2_ nanotubes could cause the movement of electrons in the valence band to the conduction band, which reduces O_2_ in the AgNPs to create •O_2_^−^ and OH• free radicals. Under visible light, the electrons on the surface of the AgNPs are excited by the surface plasmon resonance effect and move to the conduction band of the TiO_2_ nanotubes. At a concentration of 20 ppm, the TiO_2_ nanotubes alone kill *S. aureus* by 95.9% after 60 min of sunlight exposure. When AgNPs are loaded onto the TiO_2_ nanotubes, antimicrobial activity against *S. aureus* further increases to 99.99% after 60 min of sunlight irradiation. TiO_2_ NPs are capable of binding to the hydroxyl groups in the polysaccharide matrix of synthetic biofilms, resulting in irreversible adsorption [199].

*Zinc-based nanoparticles*: Similar to TiO_2_, ZnONPs exhibit broad-spectrum antimicrobial activity, mainly due to their susceptibility to ultraviolet light [200,201]. ZnO has been used as an additive in cosmetic nonprescription drug products and/or sunscreens in which the antibacterial activity is also associated with UV protection [202,203]. Other applications of ZnO include use as an antibacterial agent in food packaging [204,205,206] and in restorative dentistry tissue regeneration, where antibacterial activity is coupled to an enhancement in mechanical properties [207,208,209]. 

The antibacterial mechanism of ZnONPs is due to the physical damage of membrane and intracellular ROS production. Specifically, ZnONPs with an isoelectric point usually higher than 9 exhibit strong positive surface charges under physiologic conditions, which promotes electrostatic interactions with the negatively charged surface of the bacteria [210]. Some researchers have reported a higher bactericidal activity of ZnONPs than AgNPs against *S. mutans* [133,211]. Other studies have reported the use of Zn nanostructured with other metal NPs, such as AgNPs, or titanium porous scaffolds for bone regeneration [212,213,214]. For the latter, a proliferative increase in osteoblastic cells and an inhibitory activity towards osteoclastic bone resorption has been highlighted, in addition to bactericidal action towards both Gram-positive and Gram-negative strains [215,216].

## 6. Discussion and Final Remarks 

In the field of new antibacterial materials, NPs represent a concrete alternative to antibiotics. These nanomaterials address multimolecular biotic targets, resulting in a broad spectrum of antibacterial activity, including drug-resistant bacterial variants [217]. While a high dosage could also induce toxicity for eukaryotic cells, a low dosage, sublethal for bacteria, increases the permeability of the bacteria cell membranes and, consequently, promotes the horizontal transfer of resistance genes. Several metal (Ag, Zn, and Cu) and metal oxide (ZnO, CuO, MgO, and TiO_2_) NPs have been widely utilized in several biomedical applications due to their bactericidal abilities [218,219,220,221]. AgNPs are the most used for the antibacterial coating of medical devices [222], but their accumulation in the body and toxicity limited their use. Unlike silver, copper-based nanoparticles showed a more rapid and higher microbicidal efficacy against different types of microorganisms, such as *E. coli, V. cholera, P. aeruginosa, S. typhus, S. aureus, E. faecalis, B. subtilis* and *S. faecalis*, enhancing their range of applicability [223]. Similarly, AuNPs have been used in a wide range of antibacterial applications due to their lesser toxicity to mammalian cells, even though they have considerably lower antibacterial activity than AgNPs [224]. ZnONPs possess antimicrobial activity against numerous Gram-positive (*S. aureus, S. epidermis, B. subtilis, B. cereus, L. monocytogenes, E. faecium*) and Gram-negative (*P. aeruginosa, E. coli, K. pneumoniae, Salmonella sp.*) bacteria, stimulating a considerable range of antimicrobial applications, including the food packaging industry [225].

These above-reported studies suggest that NPs are paving way as probable alternatives to antibiotics for future therapies in nanomedicine. 

Advancements in this area involve the chemical functionalization of NPs with biomolecules such as peptides, oligomers, oligonucleotide fragments, and engineered phages to address the pharmacokinetics of a given nanomaterial towards a designated target.

## Data Availability

Not applicable.
